# Antibiotic Resistance Profiles of *Escherichia coli* Recovered from Mesenteric Lymph Nodes of Free-Ranging Game Ungulates in Western Romania

**DOI:** 10.3390/antibiotics15050475

**Published:** 2026-05-07

**Authors:** Răzvan-Tudor Pătrînjan, Adriana Morar, Cristina Mirabela Gașpar, Sebastian-Alexandru Popa, Alexandra Ban-Cucerzan, Bianca Ghițan, Daiana-Ionela Cocoș, Kálmán Imre

**Affiliations:** 1 Department of Animal Production and Veterinary Public Health, Faculty of Veterinary Medicine, University of Life Sciences “King Mihai I” from Timișoara, Calea Aradului 119, 300645 Timișoara, Romania; razvan.patrinjan.fmv@usvt.ro (R.-T.P.); adrianamorar@usvt.ro (A.M.); cristina.gaspar@usvt.ro (C.M.G.); sebastian.popa@usvt.ro (S.-A.P.); alexandra.ban-cucerzan@usvt.ro (A.B.-C.); bianca.ghitan.iosud@usvt.ro (B.G.); 2 Department of Pharmacy and Pharmacology, Faculty of Veterinary Medicine, University of Life Sciences “King Mihai I” from Timișoara, Calea Aradului 119, 300645 Timișoara, Romania; daiana.cocos.fmv@usvt.ro

**Keywords:** *Escherichia coli*, antibiotic resistance, wild boar, cervids, mesenteric lymph nodes, one health, game meat safety

## Abstract

**Background/Objectives:** The emergence of antimicrobial resistance (AMR) within a One Health framework highlights the role of wildlife as environmental reservoirs. Because wild game is an increasingly important meat source, hygienic handling during evisceration is critical to prevent carcass contamination from internal tissues such as mesenteric lymph nodes (MLNs). This study aimed to investigate the occurrence and antibiotic resistance (AR) profiles of *Escherichia coli* isolated from the MLNs of hunted wild ungulates in western Romania to better understand microbiological hazards along the game meat supply chain. **Methods:** MLN samples were aseptically collected from 103 legally hunted wild boars (*Sus scrofa*, *n* = 78) and cervids (*Capreolus capreolus* and *Cervus elaphus*, *n* = 25) across two hunting grounds. *E. coli* isolation was performed utilizing selective Tryptone Bile X-Glucuronide agar. Subsequent biochemical identification and phenotypic antimicrobial susceptibility testing were conducted using the automated VITEK^®^ 2 system. **Results:** The overall *E. coli* isolation rate was 72.8% (75/103). Analyzed by host species, the bacterium was recovered from 79.4% of the sampled wild boars (62/78) and 52.0% of the cervids (13/25). Phenotypic resistance to at least one antibiotic agent was observed in 25.3% (19/75) of the isolates, most frequently against cephalosporins (cefalexin, 21.3%) and penicillins (ampicillin, 24.0%). Multidrug resistance (MDR) was identified in 20.0% (15/75) of the isolates. **Conclusions:** The detection of MDR *E. coli* phenotypes within the MLNs of free-ranging game indicates the penetration of clinically relevant resistance mechanisms into sylvatic environments. These findings underscore the potential risk of internal carcass contamination during field evisceration, highlighting the critical need for strict hygiene practices to ensure game meat safety.

## 1. Introduction

Antimicrobial resistance (AMR) in major food-borne pathogens constitutes a major and growing challenge for both human and veterinary medicine, compromising the effectiveness of antimicrobial therapies and posing a significant threat to global public health [1,2,3,4]. Resistant bacteria and their determinants can circulate among humans, domestic animals, wildlife, and environmental reservoirs, highlighting the complex and interconnected dynamics of AMR within a One Health framework [4,5,6,7]. The role of wildlife as reservoirs, vectors, and sentinels of AMR has gained increasing attention, reflecting the complex circulation of resistant bacteria across natural ecosystems [5,8,9,10,11,12,13].

Among bacteria associated with the intestinal microbiota of animals, *Escherichia coli* occupies a central role in antibiotic resistance (AR) surveillance. This species is a common commensal inhabitant of the gastrointestinal tract of humans and animals, although certain strains are capable of causing a wide range of intestinal and extraintestinal infections [14,15]. Its adaptability and capacity for the horizontal transfer of AR determinants make *E. coli* a widely used model organism for studying the distribution and evolution of AR across domestic animals, food sources, and wildlife populations [8,9,10,11,12,13,15,16,17,18].

Wild ungulates constitute an increasingly important source of meat for human consumption in many European countries, forming a growing segment of the meat market and being part of traditional diets across the continent [19,20,21]. Species such as the wild boar and several cervid species represent some of the most frequently hunted ungulates in Europe and contribute substantially to the supply of game meat entering the human food chain [22,23]. Within free-range species, the wild boar has become a subject of major concern owing to its invasive nature and significant population overgrowth. Although wild animals are rarely exposed to direct antibiotic treatments, they may acquire resistant bacteria through environmental exposure, including contact with contaminated water sources, agricultural runoff, anthropogenic pollution, or interactions with livestock and human environments [5,6,7].

From a food safety perspective, the hygienic handling of game carcasses during hunting and field dressing is a critical step for preventing bacterial contamination. During evisceration, accidental rupture of the gastrointestinal tract or contact with contaminated tissues may facilitate the transfer of intestinal microorganisms onto carcass surfaces, potentially compromising the microbiological quality of game meat [23,24,25]. While fecal samples are commonly used for microbiological surveillance in wildlife studies, other tissues, such as mesenteric lymph nodes (MLNs), may offer additional insights into the internal dissemination of enteric bacteria within the host [11,16,20,26,27]. MLNs constitute an important component of the intestinal immune system and act as a biological barrier limiting the systemic spread of microorganisms originating from the gastrointestinal tract [28,29,30]. Therefore, the detection of enteric bacteria within MLNs is consistent with possible bacterial translocation, although accidental contamination or post-mortem migration cannot be entirely excluded [16] and, in the context of game meat production, may serve as an internal source of carcass contamination during evisceration and processing [24], thereby contributing to foodborne exposure risks.

Despite increasing attention to AR at the wildlife–environment–food interface, data on resistant bacteria in wild game animals remain limited in several European regions, including Romania [20]. A better understanding of the prevalence and spread of these bacteria in wildlife is essential to assess potential public health risks associated with the consumption of game meat.

The aim of the present study was to investigate the occurrence of *E. coli* collected from hunted wild ungulates in western Romania and to characterize the antibiotic susceptibility profiles of the recovered isolates. In addition, this study explored the potential role of MLNs as possible internal sources of carcass contamination during the processing of wild game intended for human consumption, thereby contributing to a better understanding of microbiological hazards along the game meat supply chain, from forest to fork.

## 2. Results

### 2.1. Occurrence of E. coli in Mesenteric Lymph Nodes

*E. coli* was successfully recovered from 75 out of the 103 examined mesenteric lymph node (MLN) samples, yielding an overall isolation rate of 72.8% (95% CI: 63.2–81.1%). The colonization prevalence exhibited distinct and statistically significant variations depending on the host species Χ^2^(1) = 7.23, *p* = 0.007). Wild boars (*Sus scrofa*) demonstrated a notably high carriage rate, with 79.4% (62/78; 95% CI: 68.8–87.8%) of the samples testing positive. In contrast, the isolation rate among the cervid population (*Capreolus capreolus* and *Cervus elaphus*) was significantly lower, recorded at 52.0% (13/25; 95% CI: 31.3–72.2%) (Table 1).

### 2.2. Antibiotic Susceptibility and Resistance Phenotypes

Antibiotic susceptibility testing (AST) conducted on the 75 recovered *E. coli* isolates revealed that the majority of the strains (*n* = 56; 74.7%) were entirely susceptible to all antibiotic agents tested. Phenotypic resistance to at least one antibiotic agent was observed in 25.3% (19/75) of the isolates, comprising 16 strains originating from wild boars and 3 from cervids. Intermediate resistance was also noted, specifically for tetracycline (*n* = 11; 14.7%) and ampicillin (*n* = 1; 1.3%); however, for the purpose of binary statistical comparisons, and to provide a conservative estimate of fully acquired resistance traits in this wildlife population, these isolates were grouped with the susceptible cohort. Statistical analysis revealed no significant difference in the overall rate of AR between isolates recovered from wild boars (25.8%, 16/62) and those from cervids (23.1%, 3/13). However, when evaluating the resistance profiles against individual antibiotic agents, a notable exception was observed: resistance to cefalotin was significantly higher in *E. coli* strains isolated from cervids (23.1%, 3/13) compared to those originating from wild boars (1.6%, 1/62) (*p* = 0.015). However, species-specific comparisons should be interpreted cautiously due to limited cervid sample size. For all other evaluated antibiotics, the frequency of resistance did not differ significantly between the two host species (*p* > 0.05). A detailed comparative breakdown of the resistance phenotypes between wild boars and cervids, alongside the corresponding statistical *p*-values, is presented in Table 2.

Resistance to β-lactam antibiotics constituted the most frequently observed phenotype. Specifically, resistance was highly directed toward cephalosporins, with cefalexin being the most prevalent (21.3%), followed by cefalotin (5.3%). Among penicillins, resistance to ampicillin was notably high (24.0%, 18/75). Resistance to combinations of penicillins with β-lactamase inhibitors was also detected, involving amoxicillin/clavulanic acid (8.0%) and ticarcillin/clavulanic acid (2.7%).

**Figure 1:**
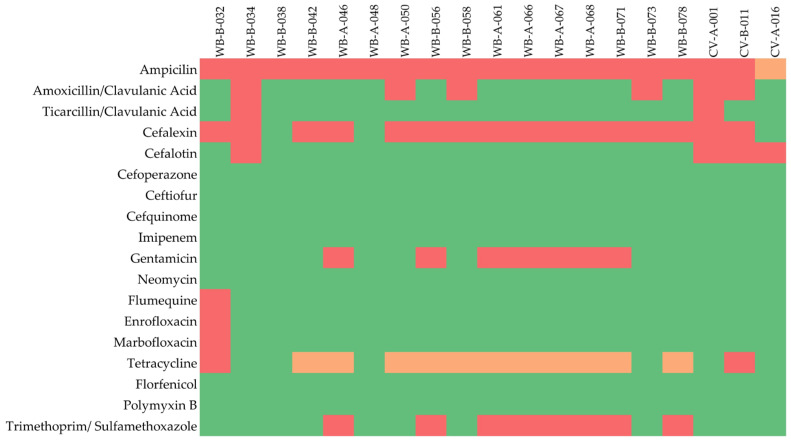
Heatmap representation of the phenotypic antibiotic resistance profiles for the 19 non-susceptible *E. coli* isolates recovered from wild boars (WB) and cervids (CV) across the two hunting grounds (A and B). Green cells indicate susceptibility (S), orange cells indicate intermediate resistance (I), and red cells represent full in vitro resistance (R) to the respective antibiotic agents.

Beyond the β-lactam class, resistance to folate pathway antagonists (trimethoprim/sulfamethoxazole) was detected in 10.7% (*n* = 8) of the isolates. Aminoglycoside resistance, exclusively directed against gentamicin, was observed in 9.3% (*n* = 7) of the strains, all of which originated from wild boars. Resistance to tetracyclines (2.7%), fluoroquinolones (enrofloxacin and marbofloxacin; 1.3% each), and quinolones (flumequine; 1.3%) remained minimal. All 75 isolates exhibited 100% susceptibility to cefoperazone, cefquinome, ceftiofur, imipenem, neomycin, florfenicol and polymyxin B. The detailed phenotypic expression of resistance is summarized in Figure 1.

### 2.3. Multidrug Resistance and MAR Index

Multidrug resistance (MDR) was identified in 20.0% (15/75) of the positive isolates. Following the international expert proposal for standardized definitions, MDR strains were recovered from both host species, including 20.9% (13/62) of the wild boar (*Sus scrofa*) isolates and 15.4% (2/13) of the cervid (*Capreolus capreolus*) isolates.

The most prevalent MDR pattern was observed in a distinct cluster of seven wild boar isolates (WB-A-046, WB-B-056, WB-A-061, WB-A-066, WB-A-067, WB-A-068, and WB-B-071), which exhibited simultaneous non-susceptibility to four antibiotic categories: penicillins (ampicillin), non-extended spectrum cephalosporins (cefalexin), aminoglycosides (gentamicin), and folate pathway inhibitors (trimethoprim/sulfamethoxazole). Additionally, complex resistance profiles involving penicillins combined with β-lactamase inhibitors (amoxicillin-clavulanic acid and/or ticarcillin-clavulanic acid) and first-generation cephalosporins were identified in two cervid isolates (CV-B-011, CV-A-001) and one wild boar isolate (WB-B-034), meeting the MDR criteria by affecting three distinct categories.

The broadest resistance spectrum was recorded for wild boar isolate WB-B-032, which was non-susceptible to agents in six distinct categories (33.3% of the 18 tested antibiotics), including fluoroquinolones and tetracyclines.

The multiple antibiotic resistance (MAR) index for the 19 non-susceptible isolates ranged from 0.05 to 0.33 (Table 3). Notably, 11 isolates presented an MAR index exceeding the 0.20 high-risk threshold, suggesting exposure to environments potentially associated with greater antibiotic selective pressure. The maximum MAR index of 0.33 was recorded for isolate WB-B-032, which demonstrated phenotypic resistance to 6 out of the 18 distinct evaluated antibiotics. The clustered MDR isolates carrying the gentamicin resistance trait uniformly yielded an MAR index of 0.22. When comparing the two host groups, neither the frequency of MDR strains (Fisher’s exact test, *p* = 0.729) nor the median MAR indices of the non-susceptible isolates (0.27 for cervids, 0.22 for wild boars) exhibited statistically significant differences (Mann–Whitney test, U = 18, *p* = 0.502), suggesting a uniform distribution of resistance traits among the circulating *E. coli* strains within this shared ecosystem.

## 3. Discussion

### 3.1. Behavioral and Ecological Drivers of E. coli Carriage and Tissue Specificity

The present study revealed a high overall *E. coli* colonization rate (72.8%, 75/103) in the MLNs of the examined wild ungulates, with wild boars acting as significantly more frequent carriers (79.4%, 62/78) compared to cervids (52.0%, 13/25). When placed in the broader European context, these tissue-specific rates offer a critical epidemiological perspective. Most prior studies have relied on traditional fecal sampling, which typically yields near-universal recovery rates, such as the 96% fecal carriage reported in a mixed population of wild ungulates from Portugal [31]. Conversely, studies utilizing alternative matrices, such as the 40.5% *E. coli* isolation rate from wild boar nasal swabs documented in Southern Italy [32], demonstrate lower colonization outside the lower gastrointestinal tract. Our 79.4% recovery from wild boar MLNs highlights that these internal nodes act as a sensitive biological trap for enteric bacteria. Consequently, the isolation of viable enteric microorganisms from these tissues suggests the occurrence of physiological bacterial translocation from the intestinal lumen, supporting the hypothesis proposed by recent tissue-specificity studies in wildlife and livestock [16,33,34]. From a game meat safety perspective, this represents a hidden risk of carcass cross-contamination during field dressing and evisceration. As highlighted by Grispoldi et al. (2020) [34] in bovine studies, lymph nodes can act as protected reservoirs for pathogens, leading to the contamination of ground meat during the technological process, even when surface hygiene is maintained. In the context of game meat consumption, our results indicate that wild ungulate carcasses may represent a potential source for resistant strains in the food chain, emphasizing the need for more stringent microbiological surveillance during the processing of wild-harvested meat.

Furthermore, the significant discrepancy in carriage rates between the two host species (79.4% vs. 52.0%) is largely driven by distinct ecological niches. Wild boars are opportunistic omnivores characterized by intensive rooting behavior, exposing them continuously to diverse environmental bacterial reservoirs, agricultural runoff, and anthropogenic waste [35]. In contrast, cervids are selective intermediate feeders that primarily browse higher vegetation, a habit that restricts their direct oral contact with contaminated soil [36]. This host-specific disparity is heavily corroborated by cross-European surveillance data. For instance, Navarro-Gonzalez et al. (2015) [37] demonstrated that in the natural environments of Spain, opportunistic wild boars exhibited notably higher *E. coli* carriage rates than sympatric selective herbivores (Iberian ibex). Similarly, a large-scale national monitoring program in Germany isolated resistant *E. coli* strains at nearly three times the rate in wild boars (6.5%) compared to roe deer (2.3%) [38]. Together, these concurrent findings from diverse geographic regions confirm the wild boar’s role as a primary, highly sensitive sentinel for environmental bacterial reservoirs, a status directly driven by their intensive rooting behavior.

### 3.2. Antibiotic Resistance Trends and Interspecies Variations

The primary concern identified in this study is the elevated level of AR in free-ranging wild ungulates, a phenomenon increasingly recognized as a significant public health threat under the One Health framework [39,40]. The highest levels of resistance in our isolates were directed toward β-lactam antibiotics, specifically cefalexin (21.3%, 16/75) and ampicillin (24.0%, 18/75). These resistance rates stand in stark contrast to the baseline resistance levels typically observed in pristine sylvatic environments. For example, nationwide monitoring in Germany demonstrated that 95.9% (210/219) of commensal *E. coli* isolated from wild boars and 97.8% (263/269) from roe deer were completely susceptible to all tested antibiotics [38]. This low baseline is further supported by Homeier-Bachmann et al. (2022) [27], who highlighted that while isolated ESBL-producing *E. coli* strains naturally exhibit complete resistance to ampicillin and other β-lactams, the overall occurrence of such resistant strains in free-ranging wildlife remains extremely low. Similarly, large-scale surveillance in Polish wild ungulates revealed that the vast majority of indicator *E. coli* belonged to the fully susceptible wild-type population; overall resistance frequencies sat at only 9.7% (27/278) for wild boars and 3.4% (6/176) for red deer, while microbiological resistance to cephalosporins was completely absent in these commensal isolates [11].

However, when wild ungulates inhabit highly anthropized environments or agricultural interfaces, their resistance burdens can far exceed our findings. Recent studies from Southern Italy report alarming resistance rates in wild boars, such as 60.5% (49/81) against ampicillin and 66.7% (54/81) against cefalexin from nasal swabs [32], alongside a massive 67.3% (138/205) overall AR rate and extreme β-lactam resistance (e.g., 67% to amoxicillin/clavulanate) from carcass swabs in the Campania region [41]. Our findings align with this geographical trend observed in Northern Italy, although with specific local variations. For instance, Mercato et al. (2022) [42] identified ESBL-producing *E. coli* in 2.6% (16/619) of samples from wild boars in the Parma province, suggesting that these animals effectively mirror the environmental resistome. However, when focusing specifically on MLNs, Bonardi et al. (2019) [16] reported a significantly lower phenotypic prevalence of only 0.9% (1/108) in the same region. The single ESBL-producing isolate identified exhibited an MDR profile, including resistance to ampicillin, amoxicillin/clavulanic acid, streptomycin, sulfasomidine, tetracycline, and trimethoprim. This places our observed 25.8% (16/62) resistance rate in an intermediate epidemiological position, confirming the broader European consensus that wild boars act as highly sensitive environmental barometers. Their opportunistic foraging behavior constantly exposes them to agricultural runoff and anthropogenic waste, making them significantly larger reservoirs for resistance compared to cervids [13,35,43]. In contrast, cervids generally present much lower resistance profiles across Europe, where resistant isolates from red deer and roe deer are consistently found at very low overall frequencies [44,45,46].

A statistically significant difference was observed regarding cefalotin resistance, which was higher in cervids. However, this specific finding must be interpreted with caution. Given that resistance to cefalexin, another first-generation cephalosporin, was comparable between the two host species, the isolated difference observed for cefalotin may partially reflect a statistical variation driven by the small sample size of the cervid cohort. Typically, wild boars exhibit higher resistance burdens across all classes compared to sympatric herbivores [38]. This specific deviation highlights complex interspecies differences and suggests that the cervids in our study area may be exposed to specific point sources of contamination. While cervids generally harbor susceptible strains, populations sharing grazing pastures with domestic livestock can acquire distinct resistant lineages. This dynamic is perfectly illustrated by Smoglica et al. (2023) [47], who evaluated a mixed cohort of wild and domestic ruminants and found phenotypic resistance to critical antibiotics exclusively in livestock and the sympatric wild ruminants sharing their pastures, while isolated wildlife remained fully susceptible.

### 3.3. The Emergence of MDR Phenotypes in the Sylvatic Environment

The identification of MDR in 20.0% (15/75) of the isolates underscores a critical public health warning. Our results mirror the established European epidemiological pattern wherein wild boars represent more significant reservoirs for MDR strains than cervids [11,48]. In our study, 13 out of the 16 wild boar strains that showed resistance were classified as MDR, a disproportionate burden consistently reflected in the literature. This trend aligns with findings from Poland [11], where wild boars showed a higher diversity of resistance compared to sympatric deer species, and recent data from Sardinia [49], which confirmed hunted wild boars as key vectors for resistant *E. coli* strains.

It is important to contextualize these findings within the broader population. The majority of the recovered *E. coli* isolates remained fully susceptible to the tested antibiotics, reflecting the baseline of a relatively preserved sylvatic environment. However, among the subset of 19 non-susceptible isolates, it is noteworthy that 11 (57.8%) yielded MAR indices exceeding the 0.20 high-risk threshold. This pattern indicates that while antibiotic resistance is not yet ubiquitous across these wildlife populations, the resistant strains that do circulate, particularly the MDR isolates, likely originate from specific point sources of intense anthropogenic contamination. The frequent exceedance of this limit within this specific subset of strains suggests localized spillover events, potentially driven by shared grazing pastures with treated livestock or agricultural run-off at the wildlife-livestock interface [50]. This intermediate position is further supported by the work of Formenti et al. (2021) [12], who reported a much higher ESBL/AmpC phenotype prevalence of 15.96% (240/1504) in wild boars from Northern Italy, indicating a greater environmental pressure than observed in our cohort. Similarly, Mercato et al. (2022) [42] found that while the overall ESBL prevalence in Parma was 2.6% (16/619), all identified ESBL-producing isolates were concurrently MDR.

### 3.4. Study Limitations

The findings of this study should be interpreted considering certain methodological limitations. First, the unequal sample sizes between wild boar (*n* = 78) and cervid (*n* = 25) groups limited the statistical power for direct interspecies comparisons and may affect the generalizability of the results. This imbalance was unavoidable due to the opportunistic sampling approach, which depended on approved hunting quotas and species availability during the respective hunting seasons. Second, the characterization of antibiotic resistance phenotypes relied solely on susceptibility testing using the automated VITEK 2 system. While this method is reliable for initial clinical and epidemiological screening, the absence of molecular confirmation (e.g., PCR targeting specific genes) limits definitive identification of the underlying resistance mechanisms. Additional limitations include the selection of a single representative colony per positive sample, which may underestimate within-host strain diversity and antibiotic resistance heterogeneity, and the inability to definitively distinguish true bacterial translocation from peri- or post-mortem contamination despite surface decontamination procedures. Finally, the lack of historical data regarding the antibiotic treatments administered to sympatric domestic livestock represents an epidemiological limitation. Access to such veterinary records would have been highly valuable to definitively trace the environmental origin of specific resistance traits and remains a key objective for further One Health investigations. Future studies incorporating whole-genome sequencing are warranted to comprehensively elucidate the molecular resistome and transmission dynamics within these wildlife populations.

## 4. Materials and Methods

### 4.1. Ethical Considerations

All procedures were limited to post-mortem sample collection from legally hunted wild animals. No live animals were handled, and all manipulations were performed in accordance with standard food safety and veterinary inspection regulations, ensuring minimal risk and full compliance with national protocols. Since the study involved exclusively post-mortem sampling of animals harvested during routine hunting activities, no additional ethical approval for animal experimentation was required.

### 4.2. Study Area

**Figure 2:**
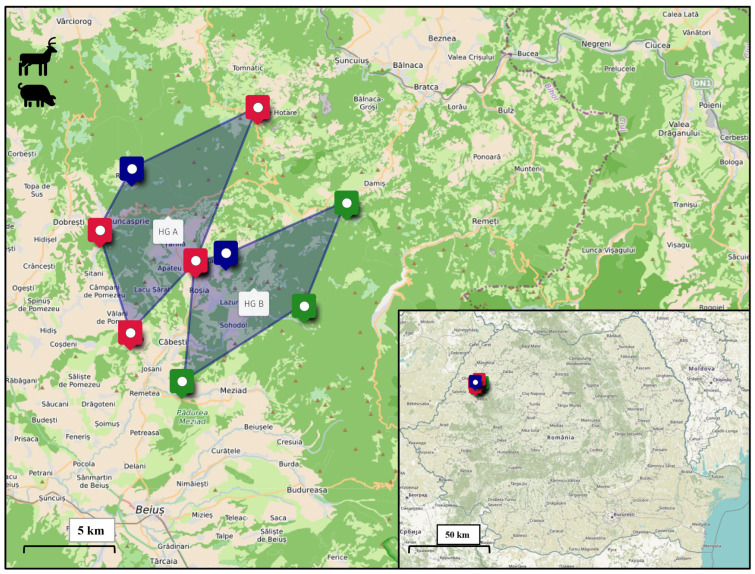
Geographical location of the study area in western Romania (Bihor County). The main map displays the boundaries of the two investigated hunting grounds, designated as HG A and HG B. Pinpoint markers indicate key territorial delineations, where red markers represent the boundaries of Hunting Ground A, green markers represent the boundaries of Hunting Ground B, and blue markers represent the designated meeting points for the hunting parties. The bottom-right inset map illustrates the regional position of the study area within the national territory of Romania.

The study area was located in Bihor County, northwestern Romania, comprising two neighboring hunting grounds (HGs) with a total surface of 20,345 hectares (ha) (Figure 2). For the purpose of this research, these areas were designated as Hunting Ground A (46°48′52.5′′ N, 22°25′17.8′′ E), and Hunting Ground B (46°52′00.9′′ N, 22°20′11.4′′ E).

The landscape in both hunting grounds is characterized by a heterogeneous mosaic of mixed deciduous and coniferous forests, interspersed with natural grasslands, small watercourses, and agricultural land. A relevant ecological feature for both areas is the significant presence of domestic livestock grazing on shared pastures. Specifically, Hunting Ground A encompasses areas used by approximately 15 cattle herds (450 head) and 3 sheep flocks (250 head), while Hunting Ground B supports 14 cattle herds (200 head) and 5 sheep flocks (300 head).

This environmental configuration creates a wildlife–livestock–human interface, where the overlap of habitats and shared water sources may facilitate the inter-species transmission of pathogens and the circulation of AR determinants within the local ecosystem.

### 4.3. Study Population and Sample Collection

The study included 103 animals: 78 wild boars (*Sus scrofa*) and 25 cervids (*Capreolus capreolus* and *Cervus elaphus*). All specimens were obtained from routine hunting activities conducted between 2024 and early 2026, in the study area.

Mesenteric lymph nodes (MLNs) were selected as the sampling tissue. Field evisceration was performed by experienced hunters, after the animals were harvested. Samples were aseptically excised immediately after evisceration, placed into sterile, labeled containers, stored at 4 °C, and transported to the laboratory on the same day. Samples were processed within 12 h to maintain bacterial viability. Individual MLN weights varied among animals, ranging from 11 to 16 g, with a mean value of approximately 15 g. Each sample was assigned a unique alphanumeric identification code indicating the animal group, hunting ground, and sampling order to ensure traceability throughout the analytical process.

### 4.4. Isolation and Preliminary Identification of E. coli

**Figure 3:**
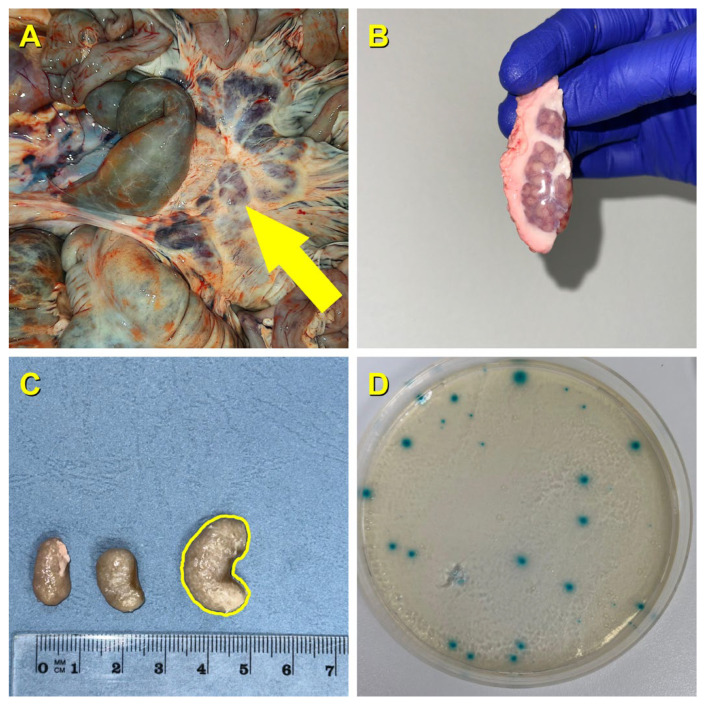
Methodological workflow from macroscopic field sampling to microbiological isolation. (**A**) Gastrointestinal tract illustrating the in situ localization of the targeted mesenteric lymph nodes. (**B**) Macroscopic aspect of an intact, aseptically excised mesenteric lymph node prior to laboratory processing. (**C**) Biometric evaluation and preparation of excised lymph nodes originating from a roe deer (*Capreolus capreolus*). (**D**) Characteristic blue-green *E. coli* colonies successfully cultured on Tryptone Bile X-Glucuronide (TBX) agar following target incubation.

To eliminate potential external contamination from field evisceration, each MLN was rinsed with sterile saline and superficially decontaminated with 70% ethanol. The nodes were allowed to air-dry completely under sterile conditions prior to aseptic homogenization to prevent residual ethanol from inhibiting bacterial growth. Following surface decontamination, the lymph nodes were aseptically sectioned, and tissue fragments of approximately 0.3 cm were obtained from the internal parenchyma using sterile scissors. A 1:9 (*w*/*v*) suspension of tissue was prepared in Buffered Peptone Water (BPW; Oxoid, Basingstoke, UK) and homogenized using a laboratory blender (Stomacher). The homogenate was then incubated at 37 °C for 18–20 h for non-selective enrichment. Following enrichment, 10 µL loopfuls were streaked onto Tryptone Bile X-Glucuronide (TBX; Biolife Italiana, Milan, Italy) agar plates. Following the principles described in ISO 16649-2:2001 [51] for *E. coli* recovery, the plates were incubated aerobically at 44 ± 1 °C for 21 ± 3 h. Colonies displaying the typical blue-green coloration were subcultured onto Brain Heart Infusion (BHI; Oxoid, Basingstoke, UK) agar. The BHI agar plates were incubated aerobically at 37 °C for 24 h to ensure the recovery of pure, isolated colonies. From each pure culture, one representative colony was selected for definitive biochemical identification and antibiotic susceptibility testing. The complete operational workflow, illustrating the progression from in situ anatomical localization of the lymph nodes to the successful cultivation of characteristic *E. coli* colonies on selective media, is detailed in Figure 3. The confirmed isolates were preserved in BHI broth supplemented with 20% glycerol and stored at −80 °C for subsequent analyses.

### 4.5. Species Confirmation and Antibiotic Susceptibility Testing

Bacterial identification and AST were performed using the VITEK^®^ 2 Compact system (bioMérieux, Marcy-l’Étoile, France). Biochemical identification was carried out using the GN identification card, whereas AST was performed with the AST-GN96 card. Minimum inhibitory concentrations (MICs) were automatically determined and interpreted according to Clinical and Laboratory Standards Institute [52] guidelines, and the European Committee on Antimicrobial Susceptibility Testing criteria [53]. The reference strain *E. coli* ATCC 25922 was used as a quality control organism for both identification and AST. Quality control procedures were performed for each run to verify the performance of culture media and AST. The AST panel comprised 18 antibiotic agents, representing multiple antibiotic classes relevant for veterinary medicine. The β-lactam group comprised ampicillin (AMP, 4–32 µg/mL), amoxicillin–clavulanic acid (AMC, 4/2–32/16 µg/mL), cefalexin (CN, 8–64 µg/mL), cefalotin (CF, 2–32 µg/mL), cefoperazone (CFP, 4–32 µg/mL), cefquinome (CEQ, 0.5–4 µg/mL), ceftiofur (CFT, 1–2 µg/mL), and imipenem (IPM, 1–12 µg/mL). Screening markers for extended-spectrum β-lactamase production included cefepime (FEP, 1 µg/mL), cefotaxime (CTX, 0.5 µg/mL), and ceftazidime (CAZ, 0.5 µg/mL), together with their respective clavulanic acid combinations (FEP/CA, 1/10 µg/mL; CTX/CA, 0.5/4 µg/mL; CAZ/CA, 0.5/4 µg/mL). The aminoglycoside class included gentamicin (GM, 4–32 µg/mL) and neomycin (N, 8–64 µg/mL), whereas the fluoroquinolone group comprised enrofloxacin (ENR, 0.25–4 µg/mL) and marbofloxacin (MRB, 1–2 µg/mL). Additional antibiotics included tetracycline (TE, 2–8 µg/mL), florfenicol (FFC, 1–8 µg/mL), polymyxin B (PB, 0.25–16 µg/mL), trimethoprim–sulfamethoxazole (SXT, 1/19–16/304 µg/mL), ticarcillin–clavulanic acid (TCC, 8/2–64/2 µg/mL), and flumequine (UB, 2–8 µg/mL).

Isolates were defined as MDR if resistant to at least one agent in three or more antibiotic classes, according to internationally accepted criteria [54]. In addition, the multiple antibiotic resistance (MAR) index was calculated for each isolate using the formula *MAR* = *a*/*b*, where *a* represents the number of antibiotic agents to which the isolate exhibited resistance and *b* is the total number of antibiotic agents tested. MAR values greater than 0.2 were considered indicative of isolates originating from environments with high antibiotic exposure [55].

### 4.6. Statistical Analysis

Data management and preliminary calculations were performed using Microsoft Excel. Prevalence was calculated as the proportion of *E. coli* positive MLNs among the total examined, with 95% confidence intervals (CI). For the comparison of *E. coli* prevalence between the two species, Pearson’s Chi-square X^2^ test was utilized. When comparing the overall AR and MDR frequencies, Fisher’s exact test (two-tailed) was applied due to the small expected frequencies in certain contingency table cells. For the purpose of binary statistical comparisons regarding antibiotic resistance, isolates exhibiting intermediate resistance phenotypes were strictly grouped with the susceptible category. The non-parametric Mann–Whitney U test was used to compare median MAR index values between species. A *p*-value of <0.05 was considered statistically significant.

## 5. Conclusions

The study demonstrates a high prevalence of *E. coli* colonization in the MLNs of free-ranging wild ungulates in western Romania, with wild boars exhibiting significantly higher carriage rates than cervids. The detection of AR, including MDR strains, highlights the penetration of clinically relevant resistance mechanisms into natural environments. The results also suggest that wild boars serve as sensitive sentinels for environmental antibiotic resistance dissemination, driven by their ecological behavior and habitat use. Overall, the findings suggest the circulation of clinically relevant resistance phenotypes in sylvatic ecosystems and highlight MLNs as a potential internal reservoir of resistant enteric bacteria that may contribute to carcass contamination during evisceration. Continued surveillance integrating molecular analyses is warranted to further elucidate resistance transmission dynamics at the wildlife–livestock–human interface and to inform effective One Health strategies.

## Figures and Tables

**Table 1:** Prevalence of *E. coli* isolated from the mesenteric lymph nodes of free-ranging wild ungulates.

Host Species	Samples (n)	Positive (n)	Prevalence (%)	95% CI (%)
*Sus scrofa*	78	62	79.4	68.8–87.8
*Capreolus capreolus*	19	10	52.6	28.9–75.5
*Cervus elaphus*	6	3	50.0	11.8–88.1
Total	103	75	72.8	63.2–81.1

**Table 2:** Comparative antibiotic resistance profiles and statistical significance of *E. coli* isolates from wild boars and cervids.

Antibiotic Agent	Wild Boar (*n* = 62)		Cervid (*n* = 13)		*p*-Value
	R	S	R	S	
Ampicillin AMP	16	46	2	11	0.504
Amoxicillin/Clavulanic acid AMC	4	58	2	11	0.582
Ticarcillin/Clavulanic acid TCC	1	61	1	12	0.318
Cefalexin CN	14	48	2	11	0.722
Cefalotin CF	1	61	3	10	0.015
Cefoperazone CFP	0	62	0	13	NA
Ceftiofur CFT	0	62	0	13	NA
Cefquinome CEQ	0	62	0	13	NA
Imipenem IMP	0	62	0	13	NA
Gentamicin GM	7	55	0	13	0.343
Neomycin N	0	62	0	13	NA
Enrofloxacin ENR	1	61	0	13	1
Marbofloxacin MRB	1	61	0	13	1
Flumequine UB	1	61	0	13	1
Tetracycline TE	1	61	1	12	0.318
Florfenicol FFC	0	62	0	13	NA
Polymyxin B PB	0	62	0	13	NA
Trimethoprim-Sulfamethoxazole SXT	8	54	0	13	0.336

**Table 3:** Resistance phenotypes, MDR status, and MAR index of the 19 non-susceptible *E. coli* isolates.

Isolate ID	Host Species	Resistance Phenotype	MDR Status	MAR Index
WB-B-032	*Sus scrofa*	AMP, CN, ENR, MRB, UB, TE	MDR	0.33
CV-B-011	*C. capreolus*	AMP, AMC, CN, CF, TE	MDR	0.27
CV-A-001	*C. capreolus*	AMP, AMC, TCC, CN, CF	MDR	0.27
WB-B-034	*Sus scrofa*	AMP, AMC, TCC, CN, CF	MDR	0.27
WB-A-046	*Sus scrofa*	AMP, CN, GM, SXT	MDR	0.22
WB-B-056	*Sus scrofa*	AMP, CN, GM, SXT	MDR	0.22
WB-A-061	*Sus scrofa*	AMP, CN, GM, SXT	MDR	0.22
WB-A-066	*Sus scrofa*	AMP, CN, GM, SXT	MDR	0.22
WB-A-067	*Sus scrofa*	AMP, CN, GM, SXT	MDR	0.22
WB-A-068	*Sus scrofa*	AMP, CN, GM, SXT	MDR	0.22
WB-B-071	*Sus scrofa*	AMP, CN, GM, SXT	MDR	0.22
WB-B-078	*Sus scrofa*	AMP, CN, SXT	MDR	0.16
WB-B-058	*Sus scrofa*	AMP, AMC, CN	MDR	0.16
WB-B-073	*Sus scrofa*	AMP, AMC, CN	MDR	0.16
WB-A-050	*Sus scrofa*	AMP, AMC, CN	MDR	0.16
WB-B-042	*Sus scrofa*	AMP, CN	Non-MDR	0.11
WB-B-038	*Sus scrofa*	AMP	Non-MDR	0.05
WB-A-048	*Sus scrofa*	AMP	Non-MDR	0.05
CV-A-016	*C. capreolus*	CF	Non-MDR	0.05

## Data Availability

Data are contained within the article.

## Abbreviations

The following abbreviations are used in this manuscript:

AMC	Amoxicillin/Clavulanic acid
AMP	Ampicillin
AMR	Antimicrobial resistance
AR	Antibiotic resistance
AST	Antibiotic susceptibility testing
BHI	Brain Heart Infusion
BPW	Buffered Peptone Water
CEQ	Cefquinome
CF	Cefalotin
CFP	Cefoperazone
CFT	Ceftiofur
CI	Confidence interval
CLSI	Clinical and Laboratory Standards Institute
CN	Cefalexin
CV	Cervid
ENR	Enrofloxacin
ESBL	Extended-Spectrum β-Lactamase
EUCAST	European Committee on Antimicrobial Susceptibility Testing
FFC	Florfenicol
GM	Gentamicin
HG	Hunting ground
IMP	Imipenem
MAR	Multiple antibiotic resistance
MDR	Multidrug resistance
MLN/MLNs	Mesenteric lymph node/Mesenteric lymph nodes
MRB	Marbofloxacin
N	Neomycin
PB	Polymyxin B
SXT	Trimethoprim-Sulfamethoxazole
TBX	Tryptone Bile X-Glucuronide
TCC	Ticarcillin/Clavulanic acid
TE	Tetracycline
UB	Flumequine
WB	Wild boar
